# Ferroptosis as an emerging therapeutic target in liver diseases

**DOI:** 10.3389/fphar.2023.1196287

**Published:** 2023-05-15

**Authors:** Yuzhen Lu, Junjie Hu, Liang Chen, Shan Li, Ming Yuan, Xianxiang Tian, Peng Cao, Zhenpeng Qiu

**Affiliations:** ^1^ College of Pharmacy, Hubei University of Chinese Medicine, Wuhan, China; ^2^ Hubei Key Laboratory of Wudang Local Chinese Medicine Research, Hubei University of Medicine, Shiyan, China; ^3^ Department of Biochemistry, Institute of Basic Medical Sciences, Hubei University of Medicine, Shiyan, China; ^4^ Department of Pharmacy, Union Hospital, Tongji Medical College, Huazhong University of Science and Technology, Wuhan, China; ^5^ Hubei Key Laboratory of Resources and Chemistry of Chinese Medicine, Hubei University of Chinese Medicine, Wuhan, China

**Keywords:** ferroptosis, nonalcoholic fatty liver disease, acute liver failure, hepatitis, hepatocellular carcinoma, liver fibrosis

## Abstract

Ferroptosis is an iron-dependently nonapoptotic cell death characterized by excessive accumulation of lipid peroxides and cellular iron metabolism disturbances. Impaired iron homeostasis and dysregulation of metabolic pathways are contributors to ferroptosis. As a major metabolic hub, the liver synthesizes and transports plasma proteins and endogenous fatty acids. Also, it acts as the primary location of iron storage for hepcidin generation and secretion. To date, although the intricate correlation between ferroptosis and liver disorders needs to be better defined, there is no doubt that ferroptosis participates in the pathogenesis of liver diseases. Accordingly, pharmacological induction and inhibition of ferroptosis show significant potential for the treatment of hepatic disorders involved in lipid peroxidation. In this review, we outline the prominent features, molecular mechanisms, and modulatory networks of ferroptosis and its physiopathologic functions in the progression of liver diseases. Further, this review summarizes the underlying mechanisms by which ferroptosis inducers and inhibitors ameliorate liver diseases. It is noteworthy that natural active ingredients show efficacy in preclinical liver disease models by regulating ferroptosis. Finally, we analyze crucial concepts and urgent issues concerning ferroptosis as a novel therapeutic target in the diagnosis and therapy of liver diseases.

## 1 Introduction

Ferroptosis is a newly identified regulated cell death (RCD) dependent on iron, first described in 2012 by Dr. Brent R Stockwell et al. (Dixon et al., 2012). This unique modality of cell death, which is distinguishable from other nonapoptotic forms of RCD according to its morphological and biochemical features, is characterized by intracellular excessive lipid peroxide accumulation and iron metabolism disturbances (Galluzzi et al., 2018). Aberrant iron accumulation, glutathione (GSH) deprivation, and glutathione peroxidase 4 (GPX4) inactivation are all critical for the onset and progression of ferroptosis (Ayala et al., 2014; Dixon and Stockwell, 2014; Yang et al., 2014; Shah et al., 2017). The regulation of ferroptosis is tightly tied to the metabolism of amino acids, lipids, and iron. Blockade of cysteine (cys) and glutamate (glu) intake involved in the synthesis of GPX4 can trigger ferroptosis by disrupting the GSH/GPX4 axis. Peroxidation of specific membrane lipids is the ultimate driver of ferroptosis (Stockwell, 2022). Arachidonic acid- (AA-) containing phosphatidylethanolamine (PE) and adrenic acid- (AdA-) containing PE were previously identified as the primary substrates for lipid peroxidation (Kagan et al., 2020), but mounting evidence raises doubts about this mechanism since a range of polyunsaturated fatty acids (PUFAs) may be implicated in ferroptosis (Zheng and Conrad, 2020). Furthermore, iron promotes ferroptosis by affecting both enzymatic and non-enzymatic processes. Iron initiates the Fenton reaction and acts as a critical cofactor in enzyme-mediated lipid peroxidation.

It has been demonstrated that various pathological processes, including malignancy (Wang et al., 2022), neurodegenerative diseases (Deas et al., 2015), pulmonary diseases (Tao et al., 2020), and liver diseases (Kim et al., 2020), are correlated with dysregulated ferroptosis. The liver, which exerts a critical effect on mammalian iron metabolism, storage, and regulation, is the primary site of iron-overload injury (Guo et al., 2021). In clinical investigations, approximately 14 mg of iron per gram of liver tissue was considered a tipping point linked with a heightened risk of cirrhosis (Adams, 2001; Angelucci et al., 2002; Powell et al., 2005; Piperno et al., 2020). Further, inhibition of ferroptosis alleviates the progression of liver diseases, such as nonalcoholic fatty liver disease (NAFLD), drug-induced liver injury (DILI), virus hepatitis, liver fibrosis, and hepatocellular carcinoma (HCC). Thus, ferroptosis may be a novel promising target for the management of liver disorders.

Herein, we outline the prominent features and molecular mechanisms of ferroptosis. Next, we focus on the relationship between ferroptosis and multiple liver diseases. Further, we highlight the therapeutic potential of ferroptosis inducers and inhibitors, including small molecular agents and natural active ingredients, for delaying the progression of liver diseases in preclinical models. Finally, we discuss and prospect the challenges and opportunities in ferroptosis applications for treating liver diseases.

## 2 The characteristics of ferroptosis

Cancer cells were usually rounded up and detached upon treatment with ferroptosis inducers (e.g., erastin) *in vitro* (Dolma et al., 2003). Effects of erastin on human prepuce fibroblast BJeLR cells were primarily focused on the alternations in mitochondrial morphology and cristae structures, including mitochondrial atrophies, increased membrane density, the reduction or even extinction of mitochondrial cristae, and the outer mitochondrial membrane (OMM) rupture. In addition, compared to other cell deaths, ferroptotic cells do not exhibit cell shrinkage, nucleus abnormality, chromatin aggregation, and disintegration of the cytoskeleton (Dolma et al., 2003; Dixon et al., 2012; Xie et al., 2016; Doll and Conrad, 2017).

Biochemically, due to intracellular GSH deprivation and GPX4 deactivation, lipid peroxides derived from Fe2+-catalyzed Fenton reaction fail to be eliminated through the GPX4-mediated reduction reaction, producing abundant reactive oxygen species (ROS), which facilitates ferroptosis (Stockwell et al., 2017; Hirschhorn and Stockwell, 2019). Numerous genes have been confirmed to genetically control ferroptosis, overexpression of which has been identified as ferroptosis indicators (Tang et al., 2021). For instance, the elevation of prostaglandin-endoperoxide synthase 2 (PTGS2) expression is one of the most common features of ferroptosis (Yang et al., 2014). Lysophosphatidylcholine acyltransferase-3 (LPCAT3) and acyl-CoA synthetase long chain family member 4 (ACSL4) are the crucial enzymes regulating PUFAs biosynthesis and remodeling by catalyzing AA to phospholipid (PL) hydroperoxide (PL-OOH, such as AA-OOH), which are considered specific biomarkers and drivers of ferroptosis (Xu et al., 2020; Chen et al., 2021). Once PL-OOH cannot be degenerated by GPX4 promptly, redundant lipid peroxides result in ferroptosis. Additionally, the essential sensor of energy equilibrium, AMP-activated protein kinase (AMPK), is a double-edged sword in ferroptosis (Tang et al., 2021). It has been demonstrated that activation of AMPK by energy stress inhibits ferroptosis. In immortalized mouse embryonic fibroblasts, glucose starvation or compounds (e.g., 2-deoxy-d-glucose) was used to induce or mimic energy stress. AMPK phosphorylation activated by energy stress phosphorylated and inactivated acetyl-CoA carboxylase inhibits the biosynthesis of PUFAs and ferroptosis (Lee et al., 2020). Reversely, AMPK-mediated phosphorylation of Beclin-1 facilitates ferroptosis by directly inhibiting the activity of cys/glu antiporter system Xc-. Mechanistically, Beclin-1 binds to solute carrier family 7 member 11 (SLC7A11), a crucial part of system Xc-, inhibiting its action and subsequent ferroptosis through phosphorylating at ser90/93/96 (Song et al., 2018). Despite the tremendous effort made to elucidate the characteristics of ferroptosis, further research is still required to illuminate its specific and detailed regulatory mechanism.

## 3 The mechanism of ferroptosis

**FIGURE 1:**
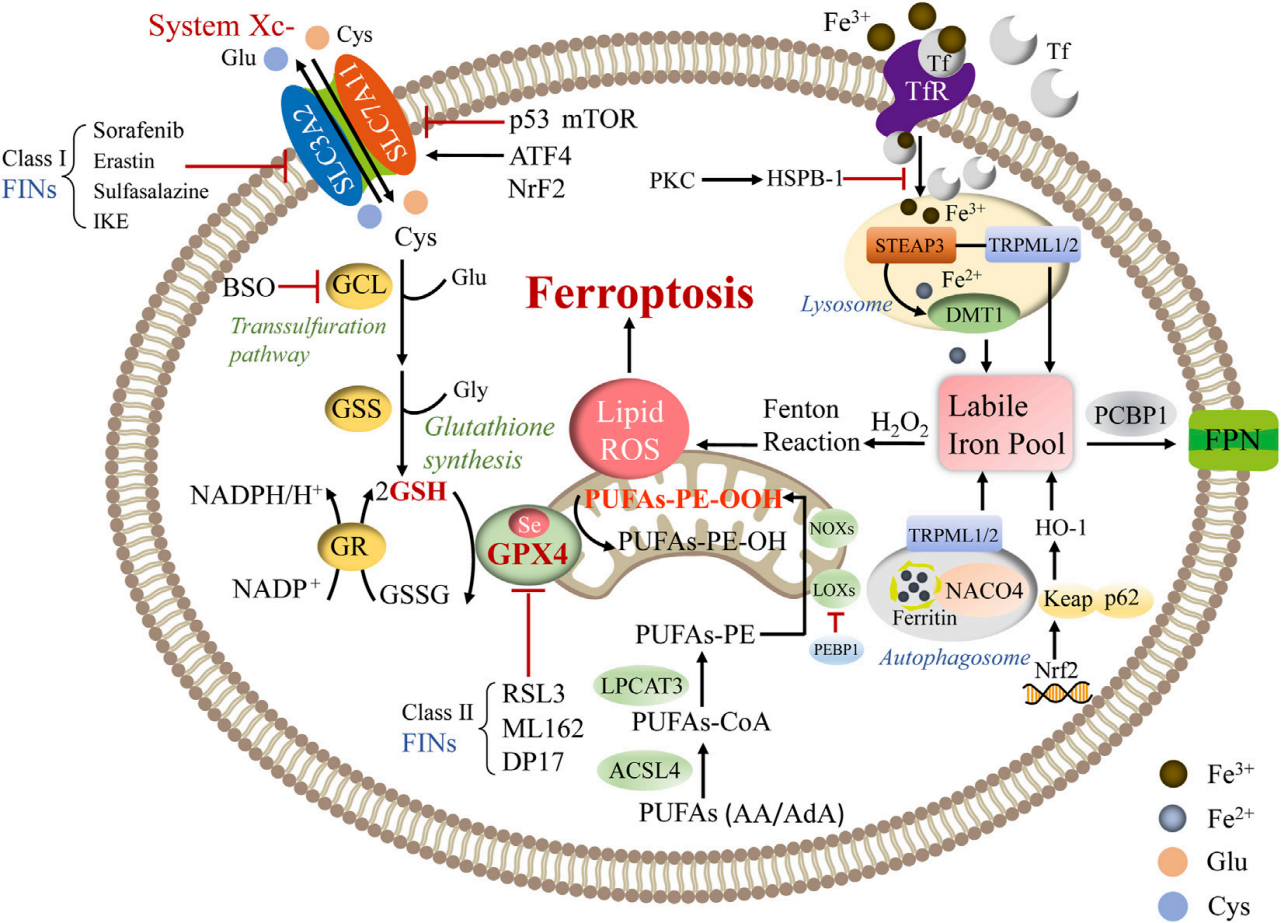
The primary regulatory mechanism of ferroptosis. Abbreviations: ATF4: activating transcription factor 4; BSO: buthionine sulfoximine; DMT1: divalent metal transporter 1; FPN: ferroportin; GCL: glutamate-cysteine ligase; GR: glutathione reductase; H2O2: hydrogen peroxide; NOXs: NADPH oxidases; PCBP1: poly rC binding protein 1; PEBP1: phosphatidylethanolamine binding protein 1; PKC: protein kinase C; RSL3: Ras-selective lethal small molecule 3; TRPML1/2: transient receptor potential mucolipin 1/2; STEAP3: six-transmembrane epithelial antigen of prostate 3.

Ferroptosis is a nonapoptotic and iron-dependent form of cell death closely related to abnormal cellular energy metabolism induced by the toxic accumulation of lipid peroxides on cellular membranes. Recently, research in the field of ferroptosis has seen exponential growth. Mounting evidence has demonstrated that ferroptosis could be initiated and implemented by assorted intracellular metabolic processes, including amino acid, lipid peroxidation, and iron metabolism (Figure 1).

### 3.1 Amino acid metabolism

Certain amino acids and derivatives stimulate ferroptosis via modulating oxidative stress. For instance, the GSH/GPX4 axis serves as a barrier to obstruct ferroptosis, while cys is the inhibitory amino acid in the synthesis of GSH and blockade of cys import via SLC7A11 triggering ferroptosis by decreasing GSH levels (Sun et al., 2018). Glu is also a decisive regulator of ferroptosis. It contributes to GSH composition and impacts the system Xc-function. The elevation of extracellular glu levels suppresses system Xc-, leading to the initiation of ferroptosis (Conrad and Sato, 2012).

#### 3.1.1 System Xc-

System Xc-is consisted of SLC7A11 (catalytic subunit) and solute carrier family 3 member 2 (SLC3A2, regulatory subunit) (Dixon et al., 2012). The system Xc-complex on the cell surface sustains redox homeostasis by absorbing external cys and swapping it for intracellular glu in a 1:1 M ratio (Sato et al., 1999). Following importation across the plasma membrane by system Xc-, cys is promptly converted to cysteine, which is considered the limiting precursor of GSH synthesis. GSH is a common endogenous antioxidant with reduced (GSH) and oxidized (GSSG) states, and the GSH/GSSG ratio indicates the degree of cellular oxidative stress (Fujii et al., 2019). Ferroptosis is enhanced by specific events that lower intracellular cysteine levels and subsequently reduce GSH contents. Further, an intricate dual transcriptional regulation governs SLC7A11. The expression of SLC7A11 is positively regulated by nuclear factor erythroid 2-related factor 2 (Nrf2) and activating transcription factor 4. Also, SLC7A11 is negatively modulated by activating transcription factor 3 and p53 at the transcriptional level (Koppula et al., 2021a). Moreover, the mammalian target of rapamycin (mTOR) complex family proteins regulates ferroptosis by manipulating SLC7A11 (Xu et al., 2021). mTORC1 enhances SLC7A11 protein stability by reducing lysosomal degradation; however, high cell density inhibits mTORC1 and promotes SLC7A11 lysosomal degradation (Koppula et al., 2021a). Additionally, mTORC2 decreases the functionality of the SLC7A11 transporter via phosphorylating SLC7A11 at serine 26 (Gu et al., 2017).

#### 3.1.2 GPX4

GPX4, a selenoenzyme, is the sole member of the PL-OOH scavenger family that convert toxic lipid hydroperoxides to nontoxic phospholipid alcohol (Ursini et al., 1985). GPX4 reduces one PL-OOH molecule to one alcohol molecule by using two GSH molecules as donors and produces GSSG, which can be converted to GSH with NADPH/H+ and glutathione reductase (Lin et al., 2020). A variety of approaches and compounds could induce ferroptosis by affecting GPX4 activity. Class I ferroptosis inducers (FINs), including sorafenib, sulfasalazine, erastin, and its analog imidazole ketone erastin, inhibit system Xc-, disturb the synthesis of GSH, and subsequently deplete GSH to suppress the GPX4 enzyme activity. Other FINs directly suppress GPX4 activity, such as ML162, DPI7, and Ras-selective lethal small molecule (RSL-)-3 (Li et al., 2020; Cui et al., 2022). Moreover, buthionine sulfoximine sensitizes cells to ferroptosis by blocking GPX4 translation, and its effect is achieved by repressing glutamate-cysteine ligase and triggering GSH depletion (Sun et al., 2018; Cui et al., 2022). Furthermore, GPX4 mediates multiple physiological processes due to its capacity to avoid cell death and maintain homeostasis in various conditions (Wortmann et al., 2013). In addition, holistic GPX4 deletion in mice resulted in embryonic death, while conditional knockout of GPX4 in mice exhibited abnormalities in multiple organs, including the brain, kidney, endothelium, and liver (Maiorino et al., 2018).

### 3.2 Lipid metabolism

Aberrant lipid metabolism may enhance lipid peroxidation and induce ferroptosis by altering the lipid compositions of biomembranes (Harayama and Riezman, 2018). In molecular dynamics models, it has been discovered that lipid peroxidation induces lipidomic alterations and damages biomembrane characteristics (formation of structured lipid holes, micellization, enhanced permeability, and increased membrane curvature), resulting in cellular dysfunction and death (Hulbert, 2010; Agmon et al., 2018). Both PUFA-containing membrane PLs and free PUFAs can be peroxidized. A redox lipidomic assay was used to determine which lipid species were favored in regulating ferroptotic cell death. Kagan et al. (2017) found four PL species, including oxygenated AA and AdA-containing PE species, are crucial for ferroptotic death signaling (C18:0/C20:4 and C18:0/C22:4). The synthesis of these two fatty acids depends on the elongation mediated by fatty acid desaturase 1 and very long chain fatty acid protein 5, both of which are increased in mesenchymal cells. Nevertheless, free PUFAs are not required for peroxidation. The bottom to switch ferroptosis is the production of coenzyme-A-derivatives of these PUFAs and their insertion into PLs (Xu et al., 2021).

ACSL4 and LPCAT3 are critical mediators of PUFA-PL synthesis, while arachidonate lipoxygenase (ALOX) is responsible for lipid peroxide synthesis (Dixon et al., 2015; Doll et al., 2017; Li et al., 2022). ACSL4 initially catalyzes the ligation of AA, with CoA to generate AA-CoA, which are subsequently re-esterified and incorporated into PE by LPCAT3 to form AA-PE. Eventually, AA-PE is oxidated to AA-OOH-PE by ALOX. ACSL proteins are mainly found in the endoplasmic reticulum (ER) and OMM. ACSLs are in charge of transforming free long-chain fatty acids into fatty acyl-CoA esters (Capelletti et al., 2020). Only ACSL4 in the ACSLs family correlates with ferroptosis and is thought to be a marker of ferroptosis sensitivity (Kagan et al., 2017). As nonheme iron-containing dioxygenases, ALOX enzymes oxidize PUFAs in a cell-type-dependent manner. Lipid peroxides are produced once ALOX catalyzes the addition of oxygen to an AA molecule. Using pharmacological suppression of ALOX subtypes under GSH depletion circumstances, Yang et al. (2016) suggested that lipoxygenases (LOXs) altered erastin-induced cell death, bolstering the notion that LOXs have an impact on ferroptosis. There are six ALOX genes in humans: ALOX5, ALOX12, ALOX12B, ALOX15, ALOX15B, and ALOXE3, which have different expression patterns in different tissues (Yang et al., 2016). Available studies have shown that various ALOX isoforms have distinctive catalytic capabilities, and experiments with isoform-specific Alox-KO mice demonstrated fundamentally varying biological functions for the different Alox isoforms. For example, the expression of ALOX15 is mediated by spermidine/spermine N1-acetyltransferase 1, a tumor protein p53 (TP53) target gene, and is involved in TP53-mediated ferroptosis (Ou et al., 2016). In contrast, ALOX12 is required for ferroptosis caused by TP53-mediated downregulation of SLC7A11 (Chu et al., 2019). However, studies on the function of ALOX isoforms in ferroptosis have so far been inconclusive. Although overexpression of isoforms such as ALOX5, ALOX12, and ALOX15 can sensitize cells to ferroptosis (Yang et al., 2022), none of the isoforms has yet been proven to play a decisive role in ferroptosis.

In addition, lipid peroxidation also occurs through nonenzymatically spontaneous autoxidations (Lee et al., 2021). In nonenzymatic autoxidations, free ferrous iron interacts with hydrogen peroxide to form ferric iron and hydroxyl radicals, which commences the process of lipid peroxidation by detaching hydrogen from the bis-allylic position of PUFAs (Reis and Spickett, 2012). Subsequently, lipid peroxidation propagates via addition, hydrogen pumping, and fragmentation, and this process is repeated to form a chain reaction (Davies and Guo, 2014). Moreover, lipid ROS triggers ferroptosis in some cell lines by activating the mitogen-activated protein kinase pathway (Yu et al., 2015; Nakamura et al., 2019). Furthermore, the metabolism of PUFAs is related to the formation of toxic byproducts, especially 4-hydroxynonenal (4-HNE), leading to the initiation of ferroptosis (Zhong and Yin, 2015).

### 3.3 Iron metabolism

Iron metabolism is inextricably linked to ferroptosis. The liver is the primary organ that controls iron metabolism because it produces and secretes hepcidin, the main iron equilibrium regulator (Ganz, 2003). Iron promotes ferroptosis by initiating the Fenton reaction, which nonenzymatically contributes to PUFA-PL peroxidation (Gaschler and Stockwell, 2017; Conrad and Pratt, 2019). Also, iron acts as a critical cofactor in enzyme-mediated lipid peroxidation (Yang et al., 2016; Koppula et al., 2021b). Enzymes that produce ROS, including ALOX, xanthine dehydrogenase, NADPH oxidase, and the mitochondrial electron transport chain complex, bind to iron, heme, or iron-sulfur clusters. Sufficient membrane PL-PUFAs and free intracellular iron are required for ferroptosis (Dixon and Stockwell, 2019). Therefore, iron chelators (e.g., deferoxamine, ciclopirox) could inhibit ferroptotic death by obstructing the production of oxidized lipid species.

The transferrin receptor (TfR) is an essential regulator of ferroptosis. Under physiological conditions, TfR on the plasma membrane regulates cellular iron absorption by transporting bound iron into cells through endocytosis (Anderson and Vulpe, 2009). TfR knockdown blocks ferroptosis production by erastin or cys depletion (Zheng and Conrad, 2020). Alternatively, once stimulated, TfR facilitated ferroptosis by replenishing the cellular iron pool (Yang and Stockwell, 2008). Additionally, iron regulatory protein 1 (IRP1) and IRP2 control iron metabolism genes, including TfR, to ensure the stability of the intercellular labile iron pool (LIP), which contains a tiny amount of free Fe2+ (Gao et al., 2015). Nuclear receptor coactivator 4 (NCOA4) is recognized as an essential receptor for the selective autophagy of ferritin. NCOA4 knockdown restricts the availability of labile iron and imparts ferroptosis resistance (Gao et al., 2016; Hou et al., 2016). NCOA4 produces ferritin and releases iron through lysosomal degeneration. Mancias et al. (2014) discovered that NCOA4 depletion hindered ferritin lysosomal localization and curtailed susceptibility to ferroptosis. The six-transmembrane epithelial antigen of prostate 3 is a metal reductase that transforms Fe3+ to Fe2+, which is then stored in a cytoplasmic LIP mediated by divalent metal transporter 1. Excessive iron in systematic circulation is reserved in ferritin, which is thought to be an iron store consisting of ferritin heavy chain 1 (FTH1) and ferritin light chain (Xie et al., 2016). Iron export is regulated by the specific ferrous iron exporter ferroportin 1 in the plasma membrane in conjunction with a multi-copper ferroxidase, such as ceruloplasmin. (Anderson and Vulpe, 2009). Iron can also be expelled as ferritin via ferritin-containing multivesicular structures and exosomes (Brown et al., 2019). Accumulation of free iron ions, which catalyze the Fenton reaction and result in lipid ROS and ferroptosis, occurs if the equilibrium among iron uptake, utilization, and recycle is disrupted. Some other iron-related proteins have also been proven to be associated with the modulation of ferroptosis, such as heme oxygenase-1 (HO-1) and poly rC binding protein 1, the latter of which is an iron chaperone in the cytoplasm that transports iron to ferritin (Bogdan et al., 2016). The chaperone action is necessary for iron-mediated cytotoxicity and ferroptosis prevention (Protchenko et al., 2021). Moreover, HO-1 promotes heme breakdown, which increases intracellular free iron levels and expedites the ferroptotic process (Chiang et al., 2018). Sun et al. (2015) reported that heat shock protein B-1 (HSPB-1) in various tumor cells was significantly inducible after erastin therapy, indicating its nonnegligible role in ferroptosis. Besides, protein kinase C phosphorylation activates HSPB-1 in HeLa cells, and HSPB-1 decreases iron levels once activated by blocking the expression of telomeric repeat binding factor 1. Furthermore, phosphorylated HSPB-1 decreases lipid peroxidation and iron absorption, suppressing ferroptosis progression. In contrast, HSBP-1 suppression exacerbates erastin-induced ferroptosis (Sun et al., 2015).

## 4 Ferroptosis in liver diseases

The liver is the metabolic hub for processing nutrients, including glucose, lipids, and amino acids. Dietary amino acids can be synthesized into various plasma proteins in the liver. The liver also governs fatty acid metabolism, with hepatocytes manufacturing and performing β-oxidation of fatty acids. Hepatic dysregulation of these nutrients results in oxidative stress and influences hepatic enzyme activities. Additionally, as the primary location of iron storage, the liver maintains iron homeostasis by generating and secreting the principal regulator of iron hepcidin (Ganz, 2003). Since oxidative stress and iron overload are significant factors in most liver diseases, ferroptosis has been involved in various hepatic pathological conditions. Of note, mounting evidence has indicated that ferroptosis of hepatocytes appears to hasten disease progression in some non-cancer liver diseases, including acute liver failure (ALF), viral hepatitis, and hemochromatosis (HH), et al. Conversely, augmented ferroptosis may sensitize aggressive forms of liver cancers (e.g., HCC, et al.) in conventional chemotherapy regimens. (Wu et al., 2021).

### 4.1 Acute liver failure

ALF is an intractable clinical syndrome with multiple causes featured by a fast reduction in hepatic function, increased aminotransferases, altered mentation, and disturbed coagulation in the absence of chronic liver diseases. Several factors facilitate ALF progression, including viral infections, improper medications, immoderate alcohol consumption, and Ischemia-reperfusion injury (IRI) (Bernal et al., 2010). More than 1,100 marketed drugs are known to cause DILI worldwide, with the nonsteroidal anti-inflammatory drug acetaminophen (APAP) being the most extensively investigated. Previous investigations have demonstrated the toxic effects of APAP metabolite N-acetyl-p-benzoquinone imine, which rapidly depletes GSH to repress mitochondrial respiration and generate ROS (James et al., 2003; Hanawa et al., 2008). Yamada et al. (2020a) have confirmed that APAP-induced ALF correlates with ferroptosis driven by ω-6 PUFAs. The ferroptosis inhibitors, such as deferoxamine (DFO), ferrostatin-1 (Fer-1), UAMC-3203, and vitamin E, exert a protective effect against this type of hepatocyte injury (Schnellmann et al., 1999; Lőrincz et al., 2015; Niu et al., 2022). In addition, lipopolysaccharide (LPS) and D-galactosamine (D-GaIN-)-induced liver injury is a widely used preclinical model of ALF. Huang et al. (2022a) found that transforming growth factor β receptor 1 (TGFβR1) was significantly elevated in LPS/D-GaIN-induced ALF and liver-specific knockout of TGFβR1-moderated LPS/D-GaIN triggered ferroptosis and apoptosis by regulating the phosphorylation of Nrf2 and glycogen synthase kinase 3β (GSK3β), and by augmenting the expression of ferroptosis-associated proteins, such as GPX4, dihydroorotate dehydrogenase, and ferroptosis suppressor protein 1 (Huang et al., 2022a). Hepatic IRI is a significant clinical problem during liver surgical procedures. A previous study has indicated that GPX4 inactivation contributes to hepatic IRI via liver injury, lipid peroxidation, and iron overload by upregulating PTGS2 (Zilka et al., 2017; Yamada et al., 2020b). Moreover, liproxstatin-1 (Lip-1) and peroxisome proliferator-activated receptor γ activator troglitazone have exhibited promising potential in preventing hepatic IRI by restraining ferroptosis (Friedmann Angeli et al., 2014; Ji et al., 2022). The latest findings further revealed that the HECT domain-containing ubiquitin E3 ligase HUWE1 was also an underlying protective modulator that could combat ferroptosis and abnormal iron accumulation by diminishing the amassing of TfR1, reducing the risk of ALF (Wu et al., 2022). However, whether TfR1 participates in ferroptosis in pathological conditions is not clearly characterized (Fillebeen et al., 2019). Therefore, researchers should conduct more in-depth and multifaceted studies to confirm that targeting the HUWE1-TfR1 axis is a new strategy for clinical intervention in ALF.

### 4.2 Viral hepatitis

Viral hepatitis is a class of infectious diseases characterized by liver inflammation and necrotic lesions induced by persistent infection of various hepatitis viruses (Guidotti and Chisari, 2006). Although hepatitis A, B, C, D, and E differ etiologically and phenomenologically, the clinical symptoms of hepatitis viruses are comparable, with hepatitis B and C being particularly linked to the development of liver fibrosis, HCC, and intrahepatic cholangiocarcinoma (Chen et al., 2021). Previous studies have indicated that the expression of miR-222 was elevated in hepatitis B virus (HBV)-infected cells. Exosomal miR-222 from HBV-infected hepatocytes triggered liver fibrosis via ferroptosis caused by TfR (Zhang et al., 2022). Further, miR-142-3p stimulates the ferroptosis in HBV-infected M1-type macrophages via SLC3A2, leading to the altered synthesis of GSH, malondialdehyde (MDA), and Fe2+ that potentially hastens the development of HCC (Hu et al., 2022).

Hepcidin is activated throughout the progression of hepatitis C. The elevation of circulating ferritin and transferrin (Tf) saturation reinforces iron storage in the hepatic cells during hepatitis C virus (HCV) infection (Wang et al., 2022). The unrestrained HCV replication results in ROS accumulation and aggravates oxidative stress in infected hepatocytes. GSK3β is essential for manipulating oxidative stress. Jiang et al. (2015) studied the GSK3β and Nrf2 signaling pathways in JFH-1 HCV-infected Huh-7.5.1 cells and biopsy liver tissue samples from hepatitis C patients. The results suggested that HCV infection triggered the antioxidative response to Nrf2, as indicated by the enhanced production of Nrf2-dependent molecule HO-1 in HCV-infected hepatocytes. Further, transforming growth factor β1 (TGF-β1) inhibits HCV-induced phosphorylation of GSK3β, which is regulated by protein phosphatase 1. Meanwhile, the response of Nrf2, the cognate substrate of GSK3β, was sharply attenuated (Jiang et al., 2015). Nevertheless, the mechanism accounting for this impaired Nrf2 response in diseased liver still needs more exploration. Hence, whether TGF-β1 interception of the Nrf2 antioxidant response in the diseased liver is the dominant pathogenic factor in its contribution to chronic liver disease still requires more rigorous study. Moreover, the GSK3β-Nrf2 signaling pathway could obstruct ferroptosis via its antioxidant potential (Huang et al., 2022a). In addition, fatty acid desaturase 2 (FADS2) is a rate-limiting element of ferroptosis since it facilitates the atypical desaturation of oleate to highly unsaturated fatty acids, especially mead acid. HCV replication is hampered by the induction of ferroptosis in host cells via FADS2 activation (Yamane et al., 2022).

### 4.3 Autoimmune hepatitis (AIH)

AIH is a progressive and chronic inflammatory liver disease possibly induced by the reciprocity of an inducement and environmental factors in a genetically vulnerable individual, affecting mainly females (Heneghan et al., 2013). Once left unattended, autoimmune hepatitis manifests a set of immune-mediated hepatic injuries toward a higher likelihood of developing end-stage liver diseases (Sucher et al., 2019). Zhu et al. observed that cyclooxygenase-2 and ACSL4 expression were elevated in the S100-mediated autoimmune hepatitis models, accompanied by the downregulated expression of FTH1 and GPX4. These findings suggest that ferroptosis could serve as a trigger or a middle mediator in the development of AIH (Adams and Barton, 2007). Obviously, further investigations are necessary to illustrate the pathological role of ferroptosis in AIH.

### 4.4 Hemochromatosis

HH is a hereditary disease manifesting excessive iron deposits in the tissues caused by autosomal recessive disorder (Pietrangelo, 2004). The development of HH is associated with mutations of the homeostatic iron regulator, which regulates the interaction between Tf and TfR (Wang et al., 2017). Due to excessive iron accumulation in parenchymal cells, ROS are generated by the Fenton reaction, resulting in oxidative damage to the body. The liver is the first to be affected (Adams and Barton, 2007). Wang et al. (2017) discovered the occurrence of ferroptosis in HH using diet-induced and transgenic mice. Meanwhile, SLC7A11 can be identified as a potential biomarker for ferroptosis in HH. It has been found that the ROS-Nrf2-antioxidant response element axis may be accountable for the elevation of SLC7A11, which is considered a potential compensatory mechanism to repress ferroptosis in HH. Although this study uncovered a possible mechanism by which ferroptosis drives HH, no explanation was given for the clinical observation that patients with type 2 HH present with symptoms earlier, develop more severe tissue damage, and have worse outcomes than patients with type 1 HH (Wang et al., 2017). In the subsequent study, researchers should focus on the different roles that ferroptosis plays in type 1 HH and type 2 HH.

### 4.5 NAFLD/nonalcoholic steatohepatitis (NASH)

NAFLD and its advanced stage, NASH, have attracted much attention due to their expanding influence on global health (Friedman et al., 2018). NAFLD is a metabolic stress-induced liver injury in which oxidative stress owing to lipid peroxide accumulation is an essential starting component. Further, iron deposition in hepatic metabolic disorders is also an exacerbating factor for NASH by aggravating the risk of hepatic inflammation and fibrosis (Yang et al., 2020; Gao et al., 2021; Ota, 2021). A high-iron diet exacerbated the oxidative and inflammatory stress in mice, hastening the NAFLD development into intractable advanced liver diseases (Videla and Valenzuela, 2022). Although the significance of ferroptosis in NAFLD and NASH remains unknown, malondialdehyde and 4-HNE, secondary lipid peroxidation products, have been widely considered indicators of oxidative stress in NASH patients (Loguercio et al., 2001). In a choline-deficient and ethionine-supplemented dietary model, the ferroptosis inhibitors reduce ferroptotic cell death and inflammatory cytokine production during the initiation stage of NASH (Tsurusaki et al., 2019). However, the investigators have not yet elucidated whether ferroptosis is involved in promoting liver tissue damage and inflammatory cell infiltration during the progressive phase of NASH. In a classic NAFLD model, Qi et al. (2020) found that ferroptosis inducer Ras-selective lethal small molecule 3 treatment exacerbated the disease symptom, including hepatic steatosis and inflammation, which could be restored by the intervention of ferroptosis inhibitors. Notably, further studies discovered an inducible GPX4 transcript-variant (iGPX4) in NAFLD conditions, and suppression of iGPX4 by siRNA-iGPX4 significantly alleviated oxidative stress and cellular damage by impairing ferroptosis. Indeed, iGPX4 may be a promising target for NAFLD therapy as activated iGPX4 interacts with the canonical cGPX4 by transforming cGPX4 from enzymatically active monomers to enzymatically inactive oligomers in response to lipid stress, which exacerbates ferroptosis (Tong et al., 2022). Thus, these findings suggested that ferroptosis regulation in the setting of NAFLD is a promising possibility as a novel therapeutic target that merits further evaluation.

### 4.6 Liver fibrosis

Liver fibrosis is a pathological alternation present in the course of most chronic liver diseases (Mederacke et al., 2015). The initiation of hepatic fibrosis is triggered by the imbalance of extracellular matrix synthesis/degradation caused by hepatic stellate cell (HSC) activation. Initiation and perpetuation are the two stages of HSC activation. The initiation phase primarily depends on paracrine mechanisms. In the sustained activation phase, HSCs exhibit cell behavior alternations, such as excessive proliferation, fibre production, and matrix disintegration (Aydın and Akçalı, 2018; Sun et al., 2020). These pathological changes disrupt the standard structure and physiological function of the liver. Thus, suppression of HSC activation has been considered a promising treatment regimen for liver fibrosis.

Recently, the function of ferritinase in the development of liver fibrosis has been revealed. Numerous genes and proteins have been identified to modulate the process of ferroptosis in HSCs. In a study concerning N6-methyladenosine (m6A) modification, Shen et al. (2021) reported that m6A modification appeared to enhance Beclin-1-mediated autophagy and consequently trigger ferroptosis in human HSCs, which is further confirmed by silencing YT521-B homology domain family, a critical m6A reader protein for sustaining Beclin-1 mRNA stability. Unfortunately, no research has been done to ascertain which selective autophagy (such as NCOA4-mediated ferritinophagy) m6A modifications affect ferroptosis (Gao et al., 2016). In addition, human umbilical cord mesenchymal stem cells promoted HSCs ferroptosis through the delivery of exosomal Beclin-1 and mediation of system Xc-/GPX4 (Tan et al., 2022). Thus, ferroptosis induction in HSCs by targeting the system Xc-/GPX4 axis might be an effective approach to ameliorate liver fibrosis.

### 4.7 Liver cancer

The prevalence of HCC has increased in recent decades (Caines et al., 2020). HCC is the fourth-leading cause of cancer death globally and the second-leading cause of years of life lost worldwide to cancer (Fitzmaurice et al., 2019; Yang et al., 2019). Current studies have confirmed that activation of ferroptosis could inhibit the disease progression of HCC in the early and advanced stages. Zhang et al. (2019) found that GPX4 was strongly expressed in fresh liver cancer tissues compared to that in paired normal liver tissues. The microarray assay results suggested that ferroptosis might be suppressed in hepatocarcinogenesis. To further confirm this hypothesis, DEN/CCl4-induced liver cancer xenograft mice were administered piperazine erastin in the presence and absence of Lip-1. They found that piperazine erastin significantly decreased the size and number of tumor foci in the liver of DEN/CCl4-induced mice and the volume of Bel-7402 cell xenografts, accompanied by reduced levels of GPX4 expression. However, Lip-1 administration abolished the antitumor effects of erastin (Zhang et al., 2019). Sorafenib is an FDA-approved drug for the treatment of patients with advanced HCC, and it has been confirmed to be a potential ferroptosis inducer. Mechanistically, sorafenib suppresses system Xc-, which promotes ER stress, GSH depletion, and the iron-dependent accumulation of lipid ROS (Dixon et al., 2014). Moreover, the p62-kelch-like ECH-associated protein 1 (Keap1)-Nrf2 pathway is associated with ferroptosis. The elevated expression of the substrate adaptor p62 protein inhibits Nrf2 degradation and promotes subsequent Nrf2 nuclear accumulation by inactivating Keap1 (Sun et al., 2016b). Of note, sorafenib controls Nrf2 to delay advanced HCC progression by regulating redox and iron metabolism.

Moreover, p53 serves as a critical mediator to affect the activity of system XC- by downregulating SLC7A11 transcription and augmenting the expression of spermine/spermidine-n1-acetyltransferase 1, raising the level of ALOX15 and boosting the accumulation of cytoplasmic peroxides (Ou et al., 2016; Zhang et al., 2019), and ultimately inducing ferroptosis in liver carcinoma cells. A growing body of surveys found that the tumor suppressor protocadherin β 14 (PCDHB14) is inactivated in the livers of HCC patients, indicating PCDHB14 may function as a potential biomarker for HCC. Notably, p53 activates PCDHB14 and inhibits SLC7A11 expression by enhancing p65 protein ubiquitination-mediated degradation, thereby abrogating p65 bind to the SLC7A11 promoter (Zhao et al., 2022). Furthermore, Wang et al. (2018) identified cystathionine β-synthase as a novel negative ferroptosis regulator using a pharmacological probe. The inhibition of cystathionine β-synthase by CH004 significantly delayed HCC growth by inducing ferroptosis *in vivo* (Wang et al., 2018). In addition, previous research revealed that loss of leukemia inhibitory factor receptor (LIFR) promoted liver tumorigenesis and conferred resistance to drug-induced ferroptosis. A lack of LIFR promotes the nuclear factor κB signaling via src homology-2 domain-containing protein tyrosine phosphatase 1, activating the iron-sequestering cytokine lipocalin 2 (LCN2), reducing iron levels, and leading to robust resistance to ferroptosis. In xenografts from HCC patients with low LIFR but high LCN2 expression, sorafenib is insensitive to suppress tumor progression by inducing ferroptosis. Hence, it might be a promising strategy to improve HCC treatment by targeting ferroptosis via anti-LCN2 medication (Yao et al., 2021). It is important to note that there is an ongoing debate on how sorafenib causes ferroptosis in HCC cells. For instance, according to research by Xiang et al. (2022), sorafenib induces ferroptosis in a manner that depends on cell density and Hippo signaling. Thus, a deeper exploration of the correlation between the various mechanisms is warranted. These findings indicate that ferroptosis may be a promising HCC therapeutic target.

## 5 Therapeutic opportunities

The induction or inhibition of ferroptosis offers considerable potential for treating several illnesses, including liver diseases. Since discovered in 2012, ferroptosis-involved studies and applications have multiplied. Numerous FINs and ferroptosis inhibitors have been uncovered, with several now in clinical trials (Stockwell, 2022). In this section, we will summarize the classical, well-recognized FINs and ferroptosis inhibitors, emphasizing their potential roles in the treatment of liver diseases. We will also point out the mechanism by which natural active ingredients halt the development of liver diseases by regulating ferroptosis (Table1).

**TABLE 1:** Promising natural active ingredient for regulating ferroptosis.

Class	Disease	Experimental model		Compound	Structure	Function	References
		*In vitro*	*In vivo*				
Ferroptosis inducers	Fibrosis	Male CCl_4_-induced chronic liver fibrosis ICR mice	Primary HSCs; LX-2	Artesunate	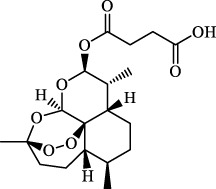	Upregulate autophagy-related genes; downregulate p62, FTH1, and NCOA4	Kong et al. (2019)
						activate ferritinophagy	
	Fibrosis	Male C57BL/KsJ-db/db mice		Artemether	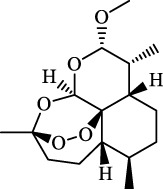	Target p53-SLC7A11; preventing the ubiquitination-mediated degradation of IRP2	Li et al. (2020b), Fu et al. (2020)
	Fibrosis	Male CCl_4_-induced liver fibrosis ICR mice	Primary HSCs; LX-2	Dihydroartemisinin	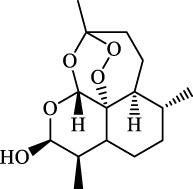	Upregulate m6A modification of BECN1 mRNA; activate ferritinophagy	Shen et al. (2022)
	Fibrosis	Male CCl_4_-induced liver fibrosis C57BL/6 mice	HSC-T6; AML-12; RAW264.7	WG	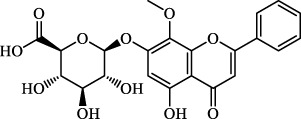	Downregulate α-SMA and COL1α1; deplete SLC7A11, GPX4, and GSH	Liu et al. (2022a)
	Fibrosis	Male CCl_4_-induced liver fibrosis C57BL/6 mice; Zebrafish raised in 0.06% TAA system water	HSC-T6	ISL	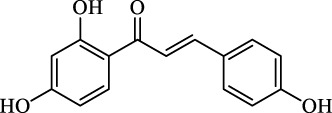	Suppress GPX4; upregulate TfR and DMT1	Huang et al. (2022b)
	Fibrosis (HBV)		HSC-T6	Chrysophanol	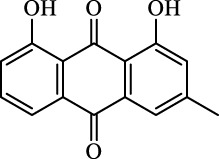	Modulate ER stress	Kuo et al. (2020)
Ferroptosis Inhibitors	ALF	Male APAP-induced DILI C57BL/6 mice	Hepa RG	(+)-CLA	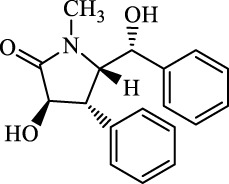	Activate Keap1–Nrf2; upregulate GPX4	Wang et al. (2020)
	ALF	Male APAP-induced DILI C57BL/6 mice	AML12	DAG	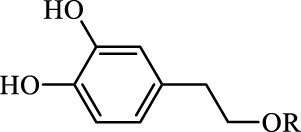	Downregulating p-ERK and HO-1; increase SLC7A11, GPX4, HO-1	Liu et al. (2022b)
	ALF	Male LPS/D-GalN-induced ALF C57BL/6 mice		tyr-Ala		Activate Keap1–Nrf2 via p62	Siregar et al. (2021)
	ALF	Male LPS/D-GalN-induced ALF C57BL/6 mice	Primary hepatocytes	Baicalin	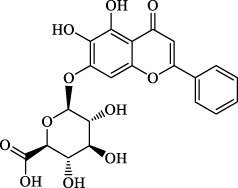	Activate Nrf2; decrease hepatic lipid deposition	Zhao et al. (2022)
	NAFLD	Male HFD-induced NAFLD C57BL/6 mice	HepG2	Ginkgolide B	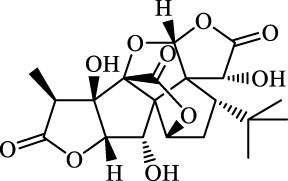	Increase HO-1, GSH, and GPX4; prevent ROS accumulation	Yang et al. (2020)
	NAFLD	Male HFD-induced NAFLD C57BL/6 mice	HEK293T; HL7702	Dehydroabietic	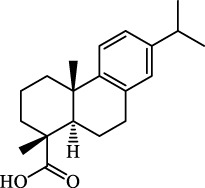	Decrease MDA	Gao et al. (2021)

Abbreviations: α-SMA: α-smooth muscle actin; COL1α1: collagen 1α1 (I).

### 5.1 Ferroptosis inducers

FINs can be categorized into four types based on diverse mechanisms (Table 2) (Feng and Stockwell, 2018). Class I FINs operate by repressing system Xc- and depleting intracellular GSH; class II FINs directly inactivate GPX4; class III FINs primarily devour GPX4 and endogenous antioxidant CoQ10; class IV FINs induce ferroptosis by exacerbating iron overload or activating HO-1 (Hassannia et al., 2019). Malignant tumors have a higher requirement for iron to support growth than normal cells, making tumor cells more vulnerable to ferroptosis (Hassannia et al., 2019). Thus, FINs raise great hopes for the potentiality of ferroptosis as a viable approach to eliminate therapy-resistant malignancies. Several FINs have been licensed by the FDA to be utilized in clinics, and they have shown remarkable efficacy as a revolutionary anticancer treatment for a range of carcinomas. For example, cisplatin is a platinum-based chemotherapy agent used for clinical chemotherapy, such as sarcoma, malignant epithelial tumors, lymphoma, and germ cell tumors (Dasari and Bernard Tchounwou, 2014). Sorafenib is the only first-line therapeutic in clinical practice for advanced HCC treatment by inducing apoptosis and impeding angiogenesis (Dixon et al., 2014; Nie et al., 2018). Louandre et al. (2013) showed that sorafenib induced ferroptosis *in vivo* (Lachaier et al., 2014; Nie et al., 2018). Also, several studies using HCC cell lines suggested that the anticancer effect of sorafenib is attributed to ferroptosis induction via SLC7A11 inhibition (Louandre et al., 2013; Dixon et al., 2014; Lachaier et al., 2014). Another study has indicated that the cell proliferation regulator retinoblastoma (Rb) protein can disturb sorafenib-induced ferroptosis in liver cancer (Louandre et al., 2015). Thus, the Rb status in HCC patients could be a tremendous prognostic marker during sorafenib therapy. Nowadays, drug resistance is a primary concern that restricts the application of sorafenib, and the average resistance impact lasts around a year. Cellular drug resistance may result from the abnormity of negatively-regulated genes of ferroptosis. Sun et al. (2016a) observed that upregulation of Metallothionein 1G (MT-1G) by Nrf2 led to resistance of HCC cells to sorafenib. In contrast, the knockdown of MT-1G enhanced lipid peroxidation and GSH depletion, contributing to sorafenib-triggered ferroptosis. Anticancer efficacy of sorafenib can be enhanced via ferroptosis by inhibiting MT-1G function (Sun et al., 2016a). In addition, Liu et al. (2021) constructed iron-based metalorganic framework nanoparticles loaded with sorafenib and a peptide with tumor-penetrating properties, synergistically delaying HCC cell malignant transformation by stimulating ferroptosis.

**TABLE 2:** Major ferroptosis inducers.

Class	Class Characteristics	Reagent	Structure	Function	Applications	References
Class I	Deplete GSH by inhibiting system Xc^-^	Erastin	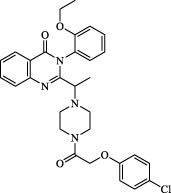	Inhibit SLC7A11; block cystine input; bind to VDAC2/3	Experimental reagent	Dixon et al. (2012)
		Piperazine erastin	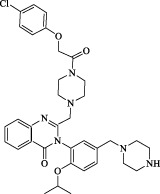	Inhibit SLC7A11; block cystine input	Experimental reagent	Yang et al. (2014)
		Imidazole ketone erastin	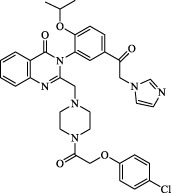	Inhibit SLC7A11; block cystine input	Experimental reagent	Zhang et al. (2019b)
		Sorafenib	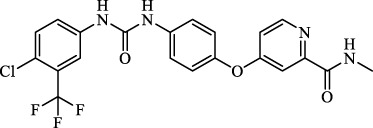	Inhibit system Xc-	Approved for marketing	Louandre et al. (2015)
		Sulfasalazine	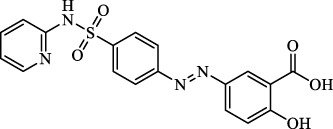	Inhibit system Xc-	Preclinical	Kim et al. (2018)
		BSO	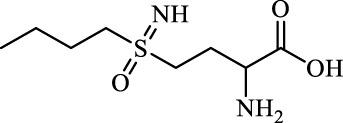	Inhibit GCL; decrease GSH synthesis	Clinical trial phase	Miess et al. (2018)
		Cisplatin	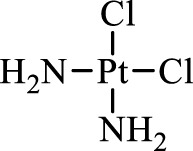	Combine with GSH to inactivate GPX4	Approved for marketing	Cheng et al. (2021)
Class II	Direct inactivate GPX4	RSL3	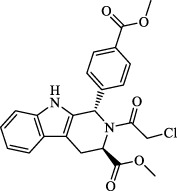 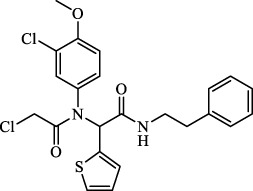	Inhibit GPX4 covalently	Experimental reagent	Yang and Stockwell (2008), Hangauer et al. (2017), Cheng et al. (2021)
		ML162/210				Weïwer et al. (2012), Yang et al. (2014)
		DPI compounds 7,10,12,13,17,18,19	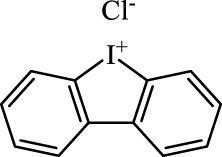	Inhibit GPX4 covalently	Experimental reagent	Yang et al. (2014)
Class III	Devour GPX4 and CoQ10 via SQS-mevalonate pathway	FIN56	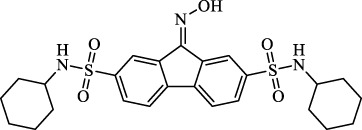	Bind and activate SQS to reduce CoQ10	Experimental reagent	Shimada et al. (2016)
		Statins	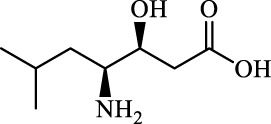	Block the synthesis of GPX4	Approved for marketing	Hung et al. (2017)
Class IV	Induct iron overload or activate HO-1	FINO2	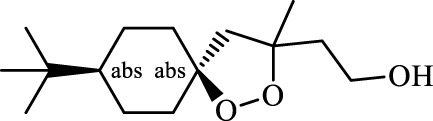	Direct oxidation of ferrous iron and lipids	Experimental reagent	Gaschler et al. (2018)
Other types		iFSP1	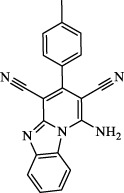	Deplete CoQ10, decrease GPX4 activity	Preclinical	Doll et al. (2019)
		MTX	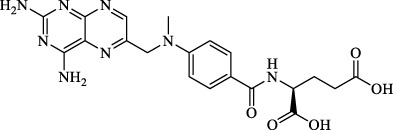	Suppress DHFR activity; inhibit BH4 production	Approved for marketing	Soula et al. (2020)
		Brequinar	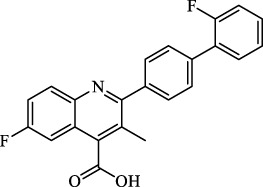	Decrease DHODH activity; cause accumulation of mitochondrial peroxide lipids	Preclinical	Mao et al. (2021)
		Dihydroartemisinin	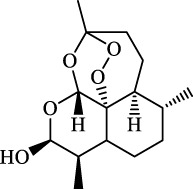	Degrade ferritin, lipid ROS	Phase II clinical trial	Zheng and Conrad (2020)

Abbreviations: SQS: squalene synthase; DHFR: dihydrofolate reductase; VDAC: voltage-dependent anion channel.

One of the crucial applications of FINs in treating liver diseases is the induction of ferroptosis in HSCs by natural active ingredients derived from traditional Chinese medicine. Artemisinin and its derivatives have strong pharmacological efficacy against liver fibrosis by elevating ferroptosis in activated HSCs. Mechanically, artesunate upregulated the expression of autophagy-associated proteins and inhibited the expression of FTH1 and NCOA4, indicating that ferritinophagy-mediated ferroptosis in HSCs is crucial in the antifibrotic therapy of artesunate (Kong et al., 2019). Further, artemether could reduce SLC7A11 expression by regulating p53, leading to ferroptosis of HSCs in fibrotic livers (Fu et al., 2020). Besides, artemether has also been found to elevate intracellular iron contents of HSCs by obstructing IRP2 ubiquitination degradation, triggering ROS accumulation and ferroptosis (Li et al., 2020). So how does artemether inhibit the ubiquitination degradation of IRP2? Li et al. (2020) discovered that STIP1 homology and U-box containing protein 1 is the E3 ligase most likely to participate in IRP2 ubiquitin, but whether another significant ubiquitinase of IRP2, F-box and Leucine Rich Repeat Protein 5, is involved in this process has not been answered positively by the researchers and needs further investigation. The stimulation of ferroptosis in HSCs by dihydroartemisinin is associated with m6A modification, which reduces fat mass by suppressing the expression of obesity-associated genes via m6A modification of BECN1 mRNA, consequently reducing liver fibrosis via ferritin autophagy-mediated ferroptosis (Shen et al., 2022). Moreover, other active ingredients of Chinese medicine, including wogonoside (WG), isoliquiritigenin (ISL), decursin, and chrysophanol, exert their antihepatic fibrosis efficacy by inducing HSCs ferroptosis. WG is an active flavonoid extracted from Scutellaria baicalensis and can effectively attenuate liver fibrosis in mice and *in vitro*. Specifically, WG-treated HSC-T6 cells showed mitochondrial ridge breakdown and condensation, and WG alleviates liver fibrosis via enhancing ferroptosis in HSCs by regulating SLC7A11, GPX4, and GSH (Liu et al., 2022). The outcomes of this study indicated that WG had a beneficial effect on liver fibrosis in mice, however to verify its therapeutic efficacy, the researchers should set up a positive control group to strengthen their conclusions. In addition, it is necessary to study data on the toxicity of WG on the liver in order to confirm the potential of WG for clinical application. Recently, Hang et al. reported that ISL alleviated liver fibrosis by triggering HSCs ferroptosis through the suppression of GPX4 and elevation of TfR and DMT1 expression, thereby generating a significant amount of ROS (Huang et al., 2022b). Another study showed that decursin administration reduced CCl4-induced hepatic fibrosis by raising Fe2+ and lipid ROS levels and decreasing GSH and GPX4 levels (Que et al., 2022). Furthermore, Kuo et al. (2020) discovered that chrysophanol from the rhizomes of Rheum palmatum L. could reduce ER stress and promote ferroptosis, which in turn mediates the activation of HSC-T6 and alleviates HBV X protein-induced liver fibrosis. Nowadays, a growing body of studies suggests that natural active ingredients may have hepatoprotective effects by augmenting ferroptosis, and more research on ferroptosis-related liver diseases is anticipated.

### 5.2 Ferroptosis inhibitors

Exogenous inhibition of ferroptosis is accomplished primarily via two approaches: iron chelation and inhibition of lipid peroxidation (Table 3) (Schnellmann et al., 1999). Some ferroptosis inhibitors are FDA-licensed or have undergone clinical trials in treating iron overload disorders. Iron chelators can chelate iron and stop the spread of lipid peroxidation by inhibiting the Fenton reaction. Numerous iron chelators have been demonstrated effective in treating ALF, NAFLD, and other non-cancer liver diseases. Schnellmann et al. (1999) discovered in 1999 that DFO might lessen APAP-induced liver damage by intracellular chelating iron. However, this change in medication hepatotoxicity is temporary. Therefore, the sustentation of DFO critical concentration is crucial for its effectiveness (Xue et al., 2016). DFO has also shown therapeutic potential in delaying the development of NAFLD and hastening the repairment of hepatic steatosis. DFO treatment alleviated hepatic iron accumulation, upregulated the expression of lipid metabolism-related proteins, and ameliorated hepatic steatosis in ob/ob NAFLD mice (Mohammed et al., 2016). Additionally, DFO possesses antifibrotic and antioxidant properties. It was discovered that DFO administration dramatically maintained liver function and reversed hepatic histopathological lesions and iron accumulation in rats. DFO also substantially reduced CCl4-induced elevation of lipid peroxidation and superoxide dismutase and GPX4 expressions.

**TABLE 3:** Major inhibitors of ferroptosis.

Class	Class Characteristics	Reagent	Structure	Function	Applications	References
Class I	Inhibit accumulation of iron	DFO	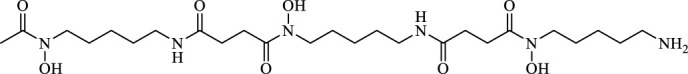	Iron consumption	Approved for marketing	Neufeld (2006), Yang and Stockwell (2008), Dixon et al. (2012), Skouta et al. (2014)
		2,2′-Bipyridine	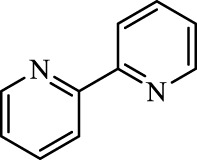		Experimental reagent	
		Ciclopirox	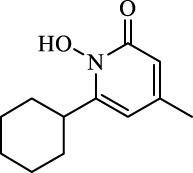		Approved for marketing	
Class II	Inhibit lipid peroxidation	Fer-1	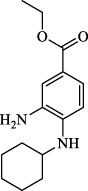	Radical scavenger	Experimental reagent	Bannai et al. (1977), Dixon et al. (2012), Friedmann Angeli et al. (2014), Hinman et al. (2018)
		Lip-1	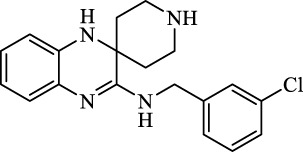		Experimental reagent	
		Vitamin E	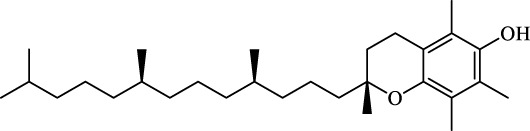		Approved for marketing	
		Rosiglitazone	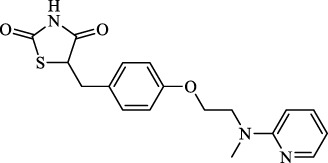	ACSL4 inhibitor; inhibit ferroptosis in a lipoxygenase-dependent manner	Approved for marketing	Shah et al. (2018)
		Troglitazone	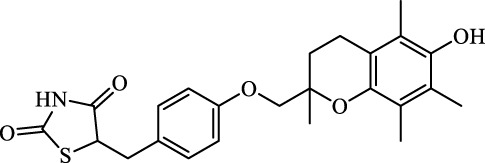		Delist	
		Pioglitazone	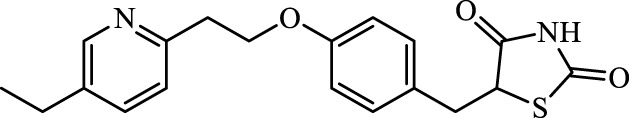		Delist	
		Necrostatin-1	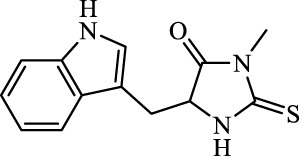	Necroptosis inhibitor; selective RIPK1 inhibitor	Preclinical	Tonnus et al. (2021)

Abbreviations: RIPK1: receptor-interacting protein kinase 1.

Lipophilic antioxidants, such as Fer-1, vitamin E, and Lip-1, act as radical scavengers to decrease lipid peroxides and are beneficial in abolishing ferroptosis to impede the progression of liver disease (Dixon et al., 2012). Fer-1 therapy was found to increase cell survival in primary mouse hepatocytes with APAP incubation, and it also showed a protective effect in other ALFs, including hepatic IRI (Lőrincz et al., 2015; Li et al., 2022). Additionally, in concanavalin A- (Con A)-induced AIH, pretreatment with Fer-1 reduced the severity of Con A-induced liver lesions and the occurrence of ferroptosis events, including the elevation of hepatic GSH, GPX4, and system Xc-expressions and downregulation of ferrous iron levels in liver tissues (Zeng et al., 2020). In addition, it was found that Vitamin E protected against excessive lipid peroxidation in GPX4-deficient mice featuring hepatic ferroptosis (Carlson et al., 2016).

Notably, multiple active ingredients extracted from natural botanicals have been demonstrated to shield the liver from ferroptosis-mediated damage. An active alkaloid (+)-clausenamide [(+)-CLA] extracted from Clausena lansium (Lour.) Skeels suppressed APAP-induced hepatocellular ferroptosis. In detail, (+)-CLA treatment reduced hepatic pathological damage and inhibited drug-induced ferroptosis by activating the Keap1-Nrf2 pathway. Mechanistically, (+)-CLA prevents Nrf2 ubiquitination and boosts Nrf2 stability by selectively interacting with the cys-151 residue of Keap1 (Wang et al., 2020). Additionally, several natural active components protect against APAP-induced DILD, such as 3,4-dihydroxyphenylethyl alcohol glycoside (DAG), which offers an alternative drug for treating ALF. Moreover, DAG extracted from Sargentodoxa cuneate exerts its antioxidant effect by upregulating GPX4 expression and downregulating phosphoextracellular regulated protein kinases and HO-1 expression to counteract APAP-induced ferroptosis in hepatocytes (Liu et al., 2022). Furthermore, it was found that oyster-derived tyr-Ala peptide showed promising hepatoprotective properties in the LPS/D-GaIN-induced ALF model by repressing ferroptotic signaling (Siregar et al., 2021). However, tyr-Ala peptide being a bioactive peptide, when administered orally they are broken down into amino acids in the gastrointestinal tract, and are less bioavailable. Therefore, to overcome this drawback, a formulation form that can be injected intramuscularly, subcutaneously or intravenously should be developed at a later stage. Moreover, Zhao et al. (2022) demonstrated that exosomes pretreated with baicalin prevent the formation of ROS and lipid peroxide-induced ferroptosis by triggering the Keap1-Nrf2 pathway via p62 and dramatically alleviating LPS/D-GaIN-induced liver injury. Simultaneously, natural active ingredients have been demonstrated to be useful in treating chronic liver diseases such as NAFLD and viral hepatitis via restraining ferroptosis. Ginkgolide B (GB) in Ginkgo biloba extracts has been reported to ameliorate hepatic lipid deposition and steatosis in obese mice via its antiferroptotic activity by activating Nrf2 (Yang et al., 2020). The authors did not, however, discuss how GB impacts high lipid levels associated with hepatic ferroptosis, and more research is required to determine the precise mechanism by which GB reduces hepatic inflammatory responses and lipid peroxidation-induced ferroptosis by activating Nrf2. Similarly, dehydroabietic acid ameliorated high-fat diet-induced NAFLD by triggering Nrf2, elevating the levels of GSH, HO-1, and GPX4 for attenuating the accumulation of ROS and lipid peroxide MDA, subsequently suppressing hepatic ferroptosis (Gao et al., 2021).

## 6 Future directions and perspectives

There is growing consensus that the aberrant activation or suppression of ferroptosis is crucial to the pathological progression of numerous liver diseases, making it a promising therapeutic target for the treatment of hepatic disorders. However, the specific role of ferroptosis has to be further studied. This review summarizes that numerous potential agents, including small-molecule compounds and natural active ingredients, may serve as ferroptosis inducers or inhibitors for treating liver diseases. Sorafenib, sulfasalazine, statins, and artemisinins have shown efficacy as monotherapy or combination therapy in treating multiple malignancies. Sorafenib is authorized to treat advanced-stage renal cell carcinoma, unresectable HCC, and differentiated thyroid carcinoma (Chen et al., 2021). Sulfasalazine has shown promise in preclinical studies as a potent inhibitor of CD133-positive and highly CD44v9-expressed HCC cells (Song et al., 2017; Wada et al., 2018).

Statins may improve the prognosis of patients with various malignancies, including HCC patients undergoing transarterial chemoembolization (Graf et al., 2008). With new technologies such as high-throughput functional screening and artificial intelligence, a growing number of ferroptosis-targeted drugs will be successfully launched.

The outcomes of ferroptosis investigations would aid in the early diagnosis of numerous liver diseases. In clinical practice, although liver function tests along with physical or imaging exams have been widely utilized as non-invasive screening methods to identify and assess different liver diseases, it is still vital to explore novel approaches to early diagnosis of liver diseases due to the lack of specificity and limitations of low sensitivity (Thapa and Walia, 2007; Chen et al., 2011). Specific ferroptosis markers have become useful diagnostic targets for various liver conditions. For instance, hepatic IRI is expected to be detected by the overexpression of the ferroptosis indicator PTGS2. Also, secondary lipid peroxidation products malondialdehyde and 4-HNE are frequently used as oxidative stress markers in NASH patients. Additionally, the levels of ferroptosis-related proteins, including GPX4, Nrf2, and Tf, can also be used to predict the onset of many liver diseases like acute and chronic hepatitis by reflecting the degree of hepatic oxidative stress and inflammation.

Further, since iron homeostasis has long been recognized to control immune system function, and lipid peroxidation has previously been linked to various immunological responses (Weismann and Binder, 2012), there is also a possibility to combine the modulation of ferroptosis with immunotherapy (Xu et al., 2021). Wang et al. identified ferroptotic regulation as a novel antitumor strategy. They claimed that augmented ferroptosis enhanced the antitumor actions of CD8^+^ T lymphocytes by stimulating ferroptosis and lipid peroxidation in tumor cells using tumor immunotherapy with a programmed death-ligand 1 (PD-L1) antibody. Further, the production of interferon-γ by CD8^+^ T lymphocytes drastically repressed the expression of SLC3A2 and SLC7A11 and the uptake of cys in the tumor cells. Additionally, the blockage of cys in conjunction with the PD-L1 antibody caused tumor cell ferroptosis and boosted T-cell immunity (Wang et al., 2019). Recently, Liu et al. (2021) synthesized a dual-targeting phosphatidylinositol-3-kinase and histone deacetylase inhibitor BEBT-908 that robustly reduces tumor cell proliferation and enhances the efficacy in antiprogrammed cell death protein 1 immunotherapy in mice by triggering immunogenic ferroptosis, demonstrating BEBT-908 would be a potential targeted therapeutic medicine against various cancer types (Fan et al., 2021). Thus, the combination of immunotherapy with ferroptosis induction could be a novel strategy in the management of liver cancers.

Altogether, a better comprehension of ferroptotic function may facilitate the discovery of efficient therapeutic approaches for therapying liver diseases, potentially bringing ferroptosis to the forefront of translational medicine in the future.
